# Assessing the best hour to start the day: an appraisal of seasonal daylight saving time

**DOI:** 10.1098/rsos.240727

**Published:** 2025-03-19

**Authors:** José María Martín-Olalla, Jorge Mira

**Affiliations:** ^1^ Departamento de Física de la Materia Condensada, Facultad de Física, Universidad de Sevilla, Sevilla ES41012, Spain; ^2^ Departamento de Física Aplicada and iMATUS, Facultade de Física, Universidade de Santiago de Compostela, Santiago de Compostela ES15782, Spain

**Keywords:** daylight saving time, summer time, sleep deprivation, motor vehicle accidents, circadian misalignment, myocardial infarction

## Abstract

We provide an evidence-based position on the seasonal regulation of clocks daylight saving time (DST) that challenges position papers by sleep associations against the practice. We review the acute, short-term impact and the chronic, long-term impact of DST in the context of the changing ambient light conditions that characterize seasons at Extratropical latitudes. We highlight the association between DST, human physiology (photoreceptive mechanisms) and human daily life. We offer a perspective on the possible scenarios should clock regulations be abandoned.

## 1. Introduction

Modern societies can be viewed as multilayer high-order networks where global synchronization mechanisms emerge [1]. One of the most visible synchronizing mechanisms is the seasonal, biannual changing of the clocks (seasonal DST, or summertime arrangements). In the near past, this issue has been subject to a strong criticism in which science, policy and society have interplayed [2]. Russia (2011), Turkey (2017), Iran (2023), Mexico (2023) and Brazil (2023), among others, recently cancelled their time arrangements. In Chile, only the most polar Región de Magallanes (2017), was able to put an end to the regulations. In the European Union (2018) and in the United States (2022) the abolish choice failed to prevail.

The changing of the clocks addresses an elusive question related to human daily cycles: the timing of the cycle or, more technically, *the phase* of the cycle. The question can be posted in a more social, global fashion: which is the best hour to start the day in view of varying sunrise times that seasons bring above approximately $30^{\circ }$ absolute latitude? The answer to this question is multifactorial but clock regulations aim to the bulk of the problem: the light conditions at work onset.

Clock regulations were proposed by Hudson [3] in 1895 and associated with seasons and latitude. Hudson’s original goal was to align social preset start times closer to sunrise, to make available longer leisure time in daylight during the spring and summer evenings. Hudson [4] described the social interplay with population groups keener to the practice and groups less favoured by it. Hudson [4] also listed energy savings as a by-effect, in line with previous thoughts by Franklin [5]. And indeed, it was not until 1916, amidst World War I, that European and American, midlatitude governments acted and adopted the practice as a war effort. Finally, Hudson [4] described the impact of the changing of the clocks in human sleep. Later, Winston Churchill summarized this issue as ‘an extra yawn some morning in April, an extra snooze time some morning in September’ [6,7].

Since the last quarter of the twentieth century [8] research studies have been able to quantify the impact of the transitions in some critical health and societal issues and to identify increase of the risks associated with the spring transition in traffic accidents [9] and heart strokes [10]. These studies describe the impact of the sudden shift of 1 h in the human circadian system. A second line of studies associates DST with a chronic misalignment, associated with the summer timing or phase of human activity, with uncertain impact on human health [11].

As a result, the practice is currently being challenged by physiologists, chronobiologists and medical doctors, chiefly sleep doctors. Position statements and position papers advocating to end the practice and lock the clocks in ST (standard time, wintertime) have been issued by the Society for Research on Biological Rhythms [12], the Sleep Research Society [13], the American Academy of Sleep Medicine [14] and the British Sleep Society [15] among others. They are conceived and written as evidence-based opinion pieces and review papers in which a myriad of research studies support the position. In this line, some research studies and review papers also support the abolition of the practice in their conclusions [9,16–19].

Understandably, these points of view have caught the attention of politicians. Governments in Europe, America and elsewhere have been pushing to cancel the regulations in the fear that they might be more harmful than initially thought. While a majority quickly embraced the idea to give away with the annoying clock transitions, the choice of the surviving clock—wintertime or summertime—is much more divisive. In addition, the preference for permanent DST still dominates polls [20], and current efforts by the circadian community try to educate people as to the consequences of permanent DST, whereas disregarding the consequences of permanent ST [17,21,22].

We maintain that these position statements and research studies only assess the cons of the practice—related to the abrupt change of one hour and to the ‘late’ summer sunsets—and pay little attention to the issues that clock regulations helped to solve, related to the alignment of work onset hours far from the sunrise, which are now forgotten. Without addressing this the balance of the risks, hazards and preferences associated with the clock regulations is not completed [23–26].

This position paper is aimed to clarify the role of the seasonal clock changing, to identify the conditions upon which modern societies find relief with such a practice, and to unveil the many misunderstandings that can be identified in medical research studies in this field. Our perspective is physical (the role of latitude), physiological (the role of ambient light conditions) and social (the role of human preferences). We will not discuss the energy savings, or the economic boost associated with the changing of the clocks [27,28]. We acknowledge that energy savings have many times prompted governments to act. Yet, the continued practice of changing the clocks cannot be easily associated with energy savings only. To put an example, since the end of World War I in 1918, Great Britain and many major cities in the USA have been practicing the seasonal changing of the clocks. The war efforts ceased, but the seasonal clock regulations continued.

## 2. What are clock regulations meant for?

Earth’s rotation is an excellent timekeeper: the time elapsed between one solar noon—or upper culmination—and the next T=24h is uniform, save for very small corrections due to Earth’s eccentricity. Uniform means independent of longitude and of latitude, and independent of calendar date. Solar noon is then the basis of time-reckoning, to which mechanical clocks are synced, the basis of preset schedules and the basis of time zones. The solar day is also the basis of the endogenous clock of many living organisms of all kinds [29,30]. This clock—the circadian system—is entrained to the sun cycle by specialized photoreceptive mechanisms [31,32]. Living organisms then exhibit rhythms or cycles that repeat day after day both at molecular basis, on an individual basis and on large-scale social environments.

Human daily rhythms entrain to the sun cycle, but they do not arrange equally around noon or midnight. Human daily sleep is typically shorter than the equatorial nighttime (T/2), meaning that humans must sustain some amount of wake at night. This occurs mainly after dusk, and seldom before dawn. This secular behaviour can be observed in pre-industrial, Tropical societies without access to artificial light where rise times usually occur at dawn, and bedtimes usually occur some 3h after the sunset [33–38]. Because of that, sunrise, instead of solar noon, is a fundamental cue for setting the timing (phase) of human cycles. The thing to note is that, unlike solar noon, sunrise is not uniform due to Earth’s axial tilt ε=23.5°=0.410rad: along a meridian sunrise times change with latitude at a given date, and at a given circle of latitude sunrise times change with calendar date; see figure S1 in electronic supplementary material. The axial tilt gives rise to variations with seasons and with latitude in human and animal circadian rhythms [39–44].

In the end, solar noon is an excellent timekeeper, but it is not so good a *phase-keeper*. In modern, Extratropical societies synchronized by preset schedules (noon), and all else equal, the seasonal changing of the clocks is nothing but a *phase selector* that imperfectly entrains to sunrise.

For a detailed description of this idea, we will make use of two locations: Bogotá (close to the Equator line) and New York (close to the 40th parallel). Both cities share the same meridian, meaning that solar noon occurs in these locations at the same time, and they share also the same mean solar time, a uniform time reference that will be used hereafter.

**Figure 1.**
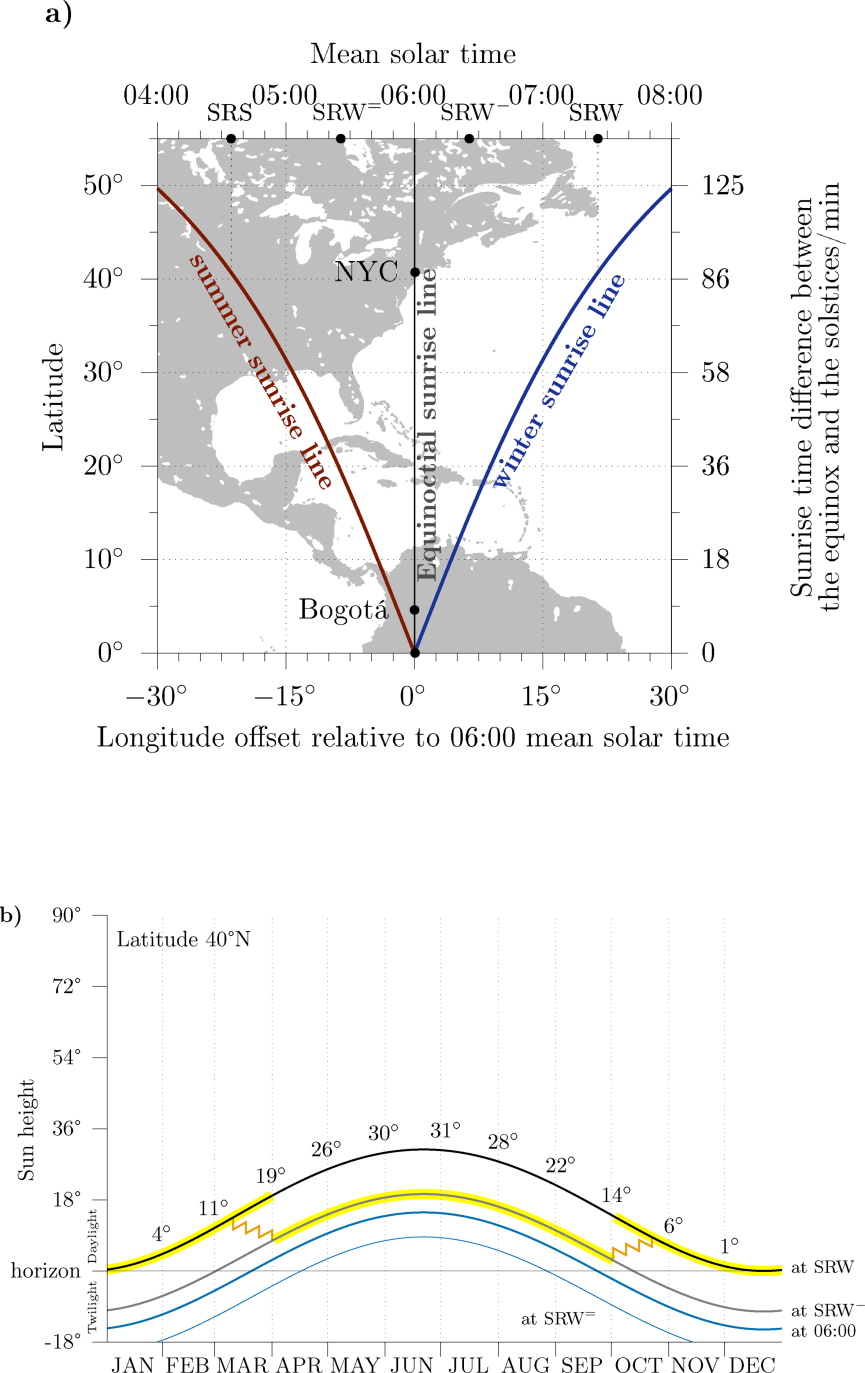
The seasonal variations of the cycle of light and dark in the morning: (*a*) the terminator line in winter and summer; (*b*) the sun height as a function of calendar date at several hours of the day. (*a*) The sunrise lines in winter (blue), summer (red) and at the equinoxes (black, vertical) with an underlying map showing America. The horizonal axes display longitude offset (bottom) and mean solar time (top, distance to solar noon). At 06.00 mean solar time the sun is about to rise (winter) or has just risen (summer) in Bogotá. In New York, the sun will rise at 07.26 (SRW, winter) or rise at 04.34 (SSS, summer). The vertical, right axis shows the lag of sunrise times relative to the sunrise time on the equinox. Earth's rotation moves the underlying map left to right at an angular speed Ω = 15° h^−1^, while the remaining layers of the panel remain still. (*b*) The evolution of sun height at SRW (black), at ${\mathrm{SRW}}^{-}$ (grey), at 06.00 (mean solar time, blue) and at ${\mathrm{SRW}}^{=}$ (thin, blue) during 1 year for 40. The regions highlighted in yellow simulate a start of the day at SRW and ${\mathrm{SRW}}^{-}$ under clock regulations. Transition dates are set in early April and early October (see §4). Note that at the Equator and at 06.00 the sun height is always zero. In either panel SRW is the winter sunrise time; ${\mathrm{SRW}}^{-}$ is 1 h before 06.26; ${\mathrm{SRW}}^{=}$ is 2 h before 05.26; SRS is the summer sunrise time. The orange zigzag lines simulate a 15min four-step adaptation before (spring) or after (autumn) transition dates.

Figure 1a shows a conventional map aligned to the Earth rotation axis (therefore aligned to clocks) and centred at the 75° W meridian, where both cities are located. The panel shows the lines of sunrise in the solstices, which are slanted, and the line of sunrise at the equinoxes (upright). Alternatively, figure S1 in electronic supplementary material shows the scenario at the solstices from a point of view aligned to Earth orbital axis so that the incoming solar radiation strikes horizontally, the line of sunrise is rendered upright, and Earth axial tilt is visible. The gradient of sunrise times is also evident. Mathematically, the sunrise time at the winter solstice (SRW) and the sunrise time at the summer solstice (SRS) are given by: [45]


(2.1)SRW(ϕ)=T2π×cos−1⁡(−tan⁡ϕtan⁡ε),(2.2)SRS(ϕ)=T2−SRW(ϕ),

where ϕ is the absolute latitude—angular distance to the Equator line, regardless of direction. For ϕ=0 (Equator line) equations (2.1) and (2.2) gives SRW(0)=SRS(0)=T/4=6h. An untilted planet (ε=0) would yield the same solution, irrespective of latitude. They break even one full revolution (T) in daylight (T/2) and nighttime (T/2).

Then, the sun rises in Bogotá around 06.00 mean solar time every day irrespective of calendar date. On the contrary, at ϕ=40° (New York) equation (2.1) yields a delay of 86min to the Equator sunrise and SRW happens at 07.26 mean solar time. Working hours that are available in Bogotá are not available in New York due to darkness.

A series of studies shows a delay in the timing of human activity with latitude following the delay in SRW.

**Figure 2.**
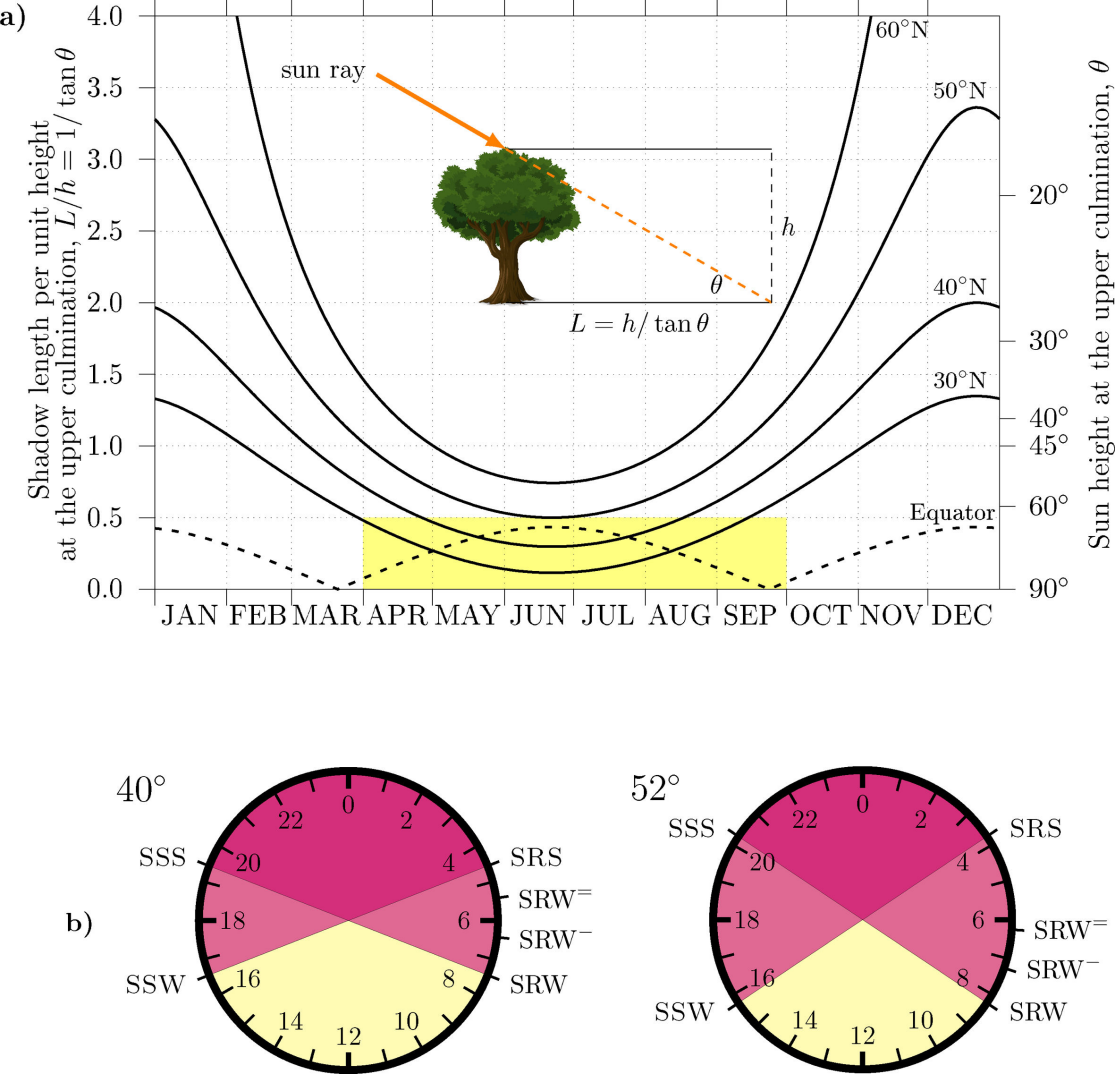
The seasonal changes around the upper culmination (*a*) and in the full day (*b*). (*a*) The sun height at the upper culmination (solar noon) $\theta =90{}^{\circ}-|\phi -\phi_{s}|$, where $\phi_{s}$ is the sun declination—the latitude of the point on the Earth with the sun straight overhead—and the shadow length per unit height length at the upper culmination 1/tan⁡θ. The yellow rectangle highlights the period of DST regulations, where insolation is characteristically tropical below 40°. (*b*) A clock with 24h analogue dial showing the permanent night (dark red), the permanent day (light yellow) and the hours of the day with day or night conditions depending on season, set for 40 latitude (above) and 52 latitude (below) [52]. In the bottom panel, SSS is the summer sunset time; and SSW is the winter sunset time.

Kung *et al*. [46] analysed morning home–work commute patterns from mobile phone data in Portugal and Ivory Coast. Like Bogotá and New York, either country is located on the same meridian and share time zone; Ivory Coast is located at the Equator, Portugal (ϕ∼40°). Kung *et al*. [46, fig. 2] show a delayed onset of commuting in Portugal, relative to the values in Ivory Coast. This delay matches with the delay in SRW.

Sani *et al*. [47] studied daily rhythmicity in 2000 individuals in Ghana (ϕ=4°), Seychelles (ϕ=5°), Jamaica (ϕ=18°), South Africa (ϕ=34°) and United States (Maywood, a suburb of Chicago, ϕ=42°). They found a delay in the acrophase—the time of the daily activity peak—with latitude that roughly matches with the delay in SRW; see electronic supplementary material, figure S2.

**Figure 3.**
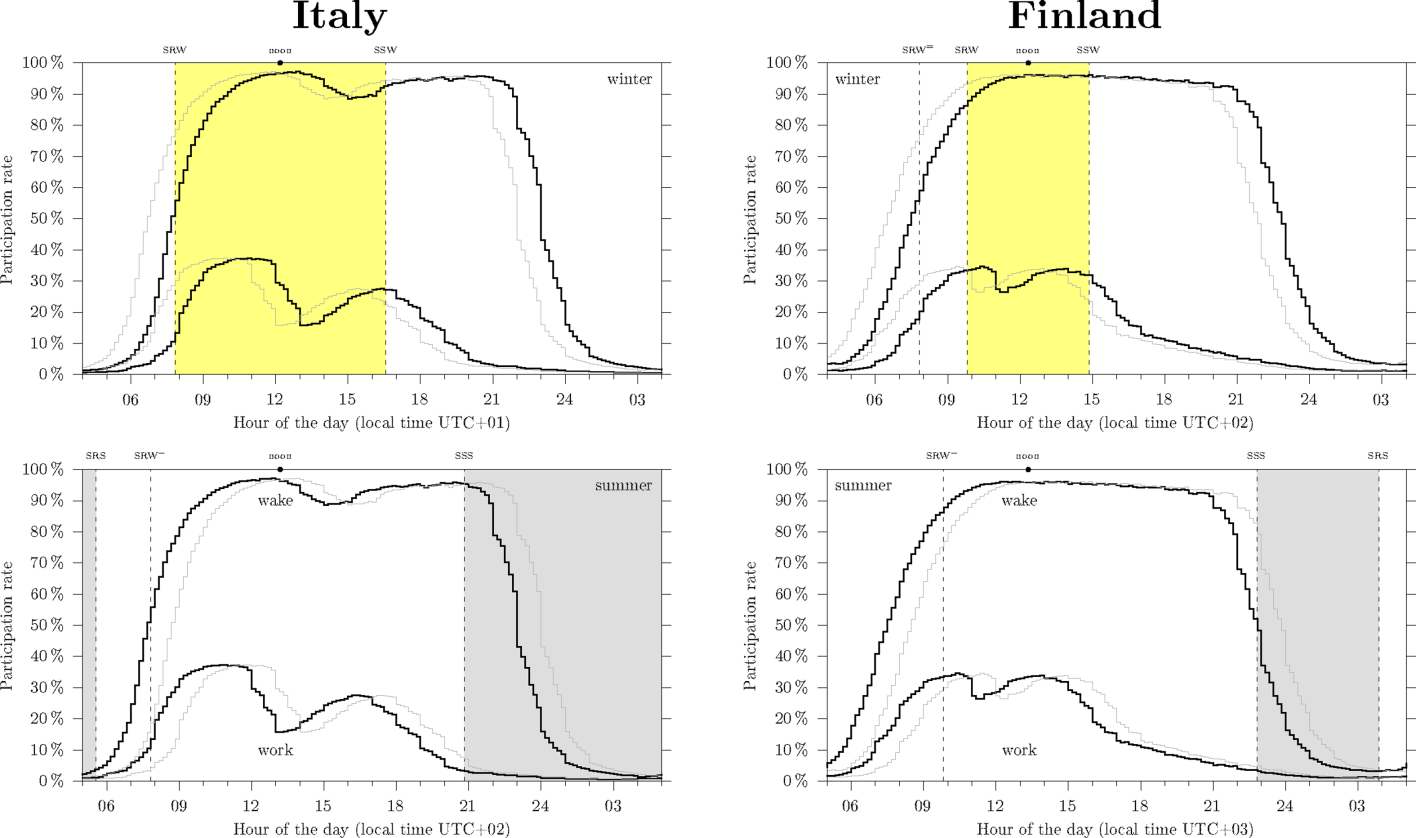
The yearly averaged wake (not sleeping and not doing personal care) participation rate and the yearly averaged work participation rate in Italy (left) and Finland (right) as obtained from the Harmonised European Time Use Survey (Hetus), black thick lines. The thin grey lines show the alternative scenario where permanent DST (top) or permanent ST (bottom) applies. The top panels annotate the winter sunrise (SRW) and the winter sunset (SSW), daytime is highlighted in yellow shade. The bottom panels annotate the summer sunrise (SRS) and the summer sunset (SSS), with nighttime highlighted in grey shade. The horizontal axes display local time. Note from top to bottom the horizontal axes are synchronous: the change in time zone is offset by a shift in the horizontal scale. In winter (top) sleep offset and work onset occur in Italy around SRW, and around ${\mathrm{SRW}}^{=}$ in Finland. Correspondingly, bedtimes in Finland come around sunset in summer (bottom), unlike in Italy. For simplicity, solar time marks are set for 43.5° N,12.3° E (Italy) and for 61, 24.9 (Finland); see Martin-Olalla & Mira [54].

Martin-Olalla [43] analysed Time Use Surveys in industrialized countries in Europe (N=18) and America (N=2) to obtain representative values of the work onset time and the sleep offset time per country. These values delayed with latitude from ϕ∼40° to ϕ∼55° following the delay of SRW [43, figs 2 and 3].

Martin-Olalla [44] conducted a review study where sleep timing in Extratropical, industrial societies (N=8) is compared with sleep timing in Tropical, pre-industrial societies with (N=5) and without (N=9) access to artificial light. Martin-Olalla [44, fig. 2 (top)] shows a synchronization of rise times and bedtimes with SRW from the Equator to ϕ∼55° during the winter season. Rise times tend to occur 1 h before SRW and bedtimes 8.5h before SRW.

Eventually, these studies show that, below the Polar Circles, the phase or timing of the sleep–wake cycle is synchronized in winter by the sunrise time (light) and not by solar noon (clocks).

Around the spring equinox the sun rises at 06.00 mean solar time irrespective of latitude. The late sunrises that lead to late awakenings in the winter vanished. In New York, the working hours that were not available in winter, due to darkness, are now available. Increasing shares of the population find benefits in harvesting these new available working hours that grow day by day until the summer solstice. We now present evidence connected with this issue.

De la Iglesia *et al*. [35] studied sleep timing in the Toba/Qom, a traditionally hunter–gatherer community living in Argentina at ϕ=23° (SRW∼06.40). The authors reported [35, table 1] earlier rise times in summer (approx. 1h) and uniform bedtimes.

Yetish *et al*. [37] reported approximately 45min earlier rise times in summer for the Tsimané people, living in Bolivia at ϕ=15° (SRW∼06.30), and uniform bedtimes. By contrast, the San people, living in Namibia desert also at ϕ=15°, show in summer 20min delayed rise times and 70min delayed bedtimes, relative to winter values.

Honma *et al*. [48] conducted a small (N=10), year-long, controlled study in Sapporo (Japan—where clock regulations are not practiced; ϕ=43°) and reported seasonal variation in the human circadian rhythm. The authors found the earliest wake-up times and the earliest bedtimes in the summer season, the latest in winter. We note that seasonal variability was larger in wake-up times (approx. 2h) than in bedtimes (approx. 1h).

Ekman [49] and van Egmond *et al*. [50] presented a case report from Uppsala, Sweden, ϕ=60° (SRW∼09.15) in the year of 1746, that showed uniform bedtimes and summer rise times 3.5h earlier than winter rise times; see also [51].

In 1810, the sessions of the Spanish National Assembly in Isla de León, ϕ=36.5° (SRW∼07.15), started 1 h earlier from May to September (09.00) than from October to April (10.00) [52,53].

The seasonal clock regulations are the current mechanism that achieves this shift towards earlier spring–summer timing in modern Extratropical societies synchronized by preset time schedules: preset start times that were associated with SRW (07.26 at ϕ=40°) are now associated with 1 h before ${\mathrm{SRW}}^{-}$ (06.26), only that, by moving clocks, a proxy is used—07.26 DST or 07.26 summertime—and the ‘same’ hour of the day remains as the starting point. The illusion brought by the shift of the clocks operates so efficiently that many, including scientists and decision makers, have forgotten their aims and goals. Compared with the 1810 case, it is just that seasonal preset schedules moved into seasonal clocks. In the end the wavy evolution of sunrise times with seasons are imperfectly sketched by a bimodal (two-season) function—late, winter; early, summer—to which the start of human activity aligns; see figure S3 in electronic supplementary material.

Figure 1b shows the evolution of the sun height at SRW and ${\mathrm{SRW}}^{-}$ at 40°N as a function of calendar date. At SRW, the sun is close to the horizon from November to February and signals the start of the day. In summer, the sun height climbs to 31° above the horizon at SRW, which challenges the role of SRW as a start of the day. Around the spring equinox, human activity can jump from SRW to ${\mathrm{SRW}}^{-}$ avoiding the dark hours of the dawn. The reverse shift can happen around the autumn Equinox with the same purpose. The region highlighted in yellow simulates the social start of the day at SRW and at ${\mathrm{SRW}}^{-}$ under clock regulations.

For reference, figure 1b also shows the sun height at 06.00, when the sun height at the Equator is always zero. At ϕ=40°, the sun height at 06.00 ranges ±15°.

The sun height is positive, and there is daylight, at ${\mathrm{SRW}}^{=}$ (2 h before SRW) from May to August. A double daylight saving time, a second change of the clocks in May, reverted in August, would move start times close to this reference point. However, this is seldom achieved for reasons that will be described later in figure 2.

Figure 2a looks at the conditions in the central hour of the day. The panel shows the yearly evolution of the sun height at solar noon θ (right axis) and the corresponding shadow length L per unit height h (left axis) for several circles of latitudes. The inset shows a visualization of the shadow of a tree. Shadow length is a common proxy for the efficiency of the solar radiation: when the sun is straight overhead the solar radiation is 100% efficient and no shadow is cast L=0. The shadow length is given by 1/tan⁡θ, with $\theta =90{}^{\circ}-|\phi -\phi_{s}|$. Here $\phi_{s}$ is the latitude of the point the Earth where the sun strikes straight upright, and no shadow is cast. For obtaining θ both ϕ and $\phi_{s}$ are signed latitudes. Figure 2a shows lower incidence angles and large shadows with increasing latitude in autumn-winter. By contrast, in spring-summer incidence angles and shadow length below 40° can be compared with those of the Equator, see the shaded area in the panel. This feature can also be perceived in electronic supplementary material, figure S1. Ignored by many, the spring–summer brings Tropical conditions below ϕ=2ε=47°.

We have completed a description of the seasonal changes in the morning, from 06.00 (see figure 1b) to 12.00 (see figure 2a). During the autumn–winter season, increasing latitude brings huge morning differences: more darkness at 06.00 and less efficient solar radiation at solar noon. As noted above, the timing of the human cycles delays with increasing latitude. During the spring–summer season, with the sun travelling at 23° latitude, mornings from the Equator to some 45° are quite more uniform. There is daylight at 06.00 and the upper culmination brings a high, Tropical insolation. Correspondingly, Martin-Olalla [44, fig. 2 (bottom)] reported uniform timing of the sleep–wake cycle in Extratropical, modern societies and in Tropical, pre-industrial societies: rise times at 05.50 and bedtimes at 21.30, mean solar times. The thing to note is that the uniformity was brought by seasonal clock regulations.

Seasonal changes at dusk are symmetrical to those described at dawn. Figure 2b shows two clocks with 24h analogue dials with the permanent night (dark red), the permanent day (light yellow) and the hours of the day with day or night condition depending on season. SRW, ${\mathrm{SRW}}^{-}$ and ${\mathrm{SRW}}^{=}$ are annotated in either clock, which is set at 40° latitude (left) and at 52° latitude (right), the latitude of London and Berlin. We note that SRW and SSS (summer sunset time) are always differ by 12h. Therefore ${\mathrm{SRW}}^{-}$ and SSS always differ by 11h. Harvesting earlier morning hours in midsummer, like ${\mathrm{SRW}}^{=}$ (see figure 1b), comes with a challenging issue: SSS differ 10h from ${\mathrm{SRW}}^{=}$, a shorter interval that can jeopardize bedtimes [26]. In the end a seasonal double DST has seldom been employed, often in connection with war times or crises (Portugal 1942−1945, and Germany 1945 and 1947).

The model in figures 1 and 2 is a simplistic description based on the goals of the regulations of clocks: ‘the alignment of work hours closer to sunrise and greater use of outdoor recreation in the [spring and summer] evening’ [6, Introduction]. Real scenarios are more complicated because, first, the onset of human activity in the morning can span several hours, displaying early risers and late risers. Early risers, those with onset times earlier than SRW, find little benefits in advancing the clocks in spring because they already have an advanced phase of activity. Late risers, those with onset times aligned to SRW find more benefits in aligning the work hours closer to sunrise in spring. Moreover, the concept of ‘onset time’ is fuzzy on an individual basis: it points to the period in which individuals resume activity after the nighttime rest: it comes from the wake-up time to the work onset, encompassing more than 1 h on an individual basis. Finally, it is not a location but an area, like a US time zone or a European country, that comes into consideration. This brings northeast (late) to northwest (early) gradients by roughly 1 h difference. Furthermore, modern access to artificial light impacts the circadian timing [55,56] and is related to modern syndromes like sleep deprivation [57] and social jet lag (SJL) [58].

Empirically, this social complexity can be circumvented by analysing time use surveys like the American Time Use Survey [59] or the Harmonised European Time Use Survey [60]. The daily rhythm of work and the daily rhythm of sleep/wake—the participation rate in the work activity and in the sleep activity—allow to assess social average work onset and sleep offset values representative of a country. Considering latitude and longitude, the distance from these time marks to the SRW can be obtained [43]. Figure 3 visualizes the scenario in Italy (left), whose median latitude is 43.5°, and in Finland (right), 61°. The panels show by black thick lines the wake participation rate and the work participation rate in winter (top) and summer (bottom) under clock regulations. In the morning, the rise of wake population and of working population are easily perceived; in the evening the dwindle of working population, later followed at night by the onset of sleep are also noted. The thin grey lines show the alternative scenario should permanent DST (top) or permanent ST (bottom) apply. Winter sunrise time and sunset time are annotated in the top panels, summer sunrise time and sunset time, in the bottom panels. The horizontal axes are synchronous from top to bottom; however, the scale is shifted to the left in the bottom panels to simulate clock regulations. Human activity entrains to local time and is then pushed to the left in the bottom panel. Note that with start work aligned to SRW in winter (Italy), bedtimes come after SSS in summer even after clock regulations apply.

## 3. How hazardous are transition dates?

In 2015, the experts summoned by the European Commission to revisit the application of summertime tended to agree in the discussion that ‘the issue that caused inconvenience was the changing of the clocks rather than the application of summertime [and wintertime] *per se*.’ [2, p. 16]. The size of the changing of the clocks (1 h) is associated with the preference for whole hours in preset schedules. As an early case, we noted in §2 the scheme followed by the Spanish National Assembly in 1810: 09.00 (May to September), 10.00 (October to April). One hour is then the smallest change in preset schedules for practical purposes. A smooth change in preset time schedules that would accommodate the smooth advance of sunrise times after the winter solstice, and the smooth delay of sunrise times after the summer solstice is unpractical [61].

All else equal, transition dates bring an abrupt change of 1 h in the phase of human cycles. Many research studies have quantified the acute or short-term effects of this change in a wide variety of health and societal issues since Monk & Folkard [8]. These studies benefit from the natural experiment that the transition dates set: the researchers find the appropriate database, accumulate as many years of observations as possible to increase sample size, and crunch the numbers to get usually observed versus expected ratios for one week or two following a transition. Results are often disaggregated by day of the week.

This methodology has been conducted in almost every societal issue from traffic accidents [9,62–70], acute myocardial infarction (AMI) [10,71–78], all-cause mortality [79–81], and a vast collection of health and societal issues like trauma admissions [82], life satisfaction [83], suicide rates [84], spontaneous pregnancy loss in *in vitro* fertilization [85], missed medical appointments [86], visits for mental health disorders [87], autopsy rates [88], spontaneous deliveries [89], white-tailed deer−vehicle collision rates [90], femur fractures in older population [91] and marathon run performance [92].

AMI studies and traffic accident studies yield the major of concerns due to their ubiquity and prevalence. The two largest studies in this field analysed the full record of fatal traffic accidents in USA (1996−2017, population 310 million) [9] and the full record of AMI events in Sweden (1987−2006, population 9 million) [10] with similar samples sizes (10 000 of counts per week), and similar results: 1.05 incidence rate ratio (IRR) in the week after the spring transition.^1^ Interestingly, Janszky & Ljung [10] reported a decrease of AMI following the autumn transition. The important epidemiological question of the net balance of the practice is yet unknown: IRR does not behave well under addition of different sets. We also note that since 2008 (16 years ago) results on Sweden AMI strokes have not been updated. Likewise, no results from a similar or larger population bracket are available.

In epidemiology, the importance of a deviation is assessed by comparing it with the standard deviation of the signal (how much the tested quantity changes from year to year in normal weeks). The ratio of these two quantities is the *z*-score [93]. Studies in this field have not addressed this quantity previously. However, in few cases it can be inferred from published data [94]. Fritz *et al*. [9, fig. 1A] shows the standard deviation of the record of weekly fatal motor vehicle accidents in the USA. The relative standard deviation (RSD) can be assessed as approximately 15% [95]. Therefore, the *z*-score of the IRR attributed to DST is roughly z∼5/15=1/3. This means that the impact of the spring transition is systematic but, also, it is slight, compared with the myriads of confounding factors that impact traffic accidents. Smaller studies can yield larger IRR with larger confidence intervals, but their RSD would also be large, and the *z*-score would remain low.

Finally, we have brought attention to the fact that transition dates are predictable. Hence, the short-term effects of the spring transition associated with sleep loss can be minimized, on an individual basis, by a circadian preadaptation [24]. As an example, individuals can set their alarm clocks 15min earlier three weeks before the spring transition date. Set the clock 15min minutes earlier two weeks, and one week before the transition; and reset the alarm clock to their regular value after the transition. The individual would complete a 4-step, 15min transition, smoother than the clock regulations (see zigzag lines in figure 1*b*). Chances that population groups would behave like that are negligibly small, because people abhor a change in their regular rhythms. Nonetheless, for those worried by the impact of the 1-h, one-step change it is a sensible choice that could be easily preset in cellulars as an option.

## 4. Setting transition dates

Clock regulations inevitably need two transition dates associated with seasons. In the past years, the layout of transition dates has received little focus. We offer here a perspective.

Transition dates currently come in late March and late October (30 DST weeks versus 22 ST weeks) in Europe. In America, they are set in early March and early November (34/18). In Chile, they come in early April and early September (30/22 as in Europe). Finally, in Australia and New Zealand they occur in early April and early October, breaking even the year (26/26).

By contrast, the layout by the Spanish National Assembly in 1810 yielded 21/31 and in the 1920s United Kingdom settled to a 23/29 layout (mid-April to early October). Globally speaking, since the inception of the practice in 1916, the ST period is shrinking little by little. On the one hand, this brings higher risks, and higher discomfort, to the population that faces a dark morning after the onset of DST, and before the onset of ST. On the other hand, the enlargement of the DST period is showing a smaller preference for ST and a higher prevalence of late risers.

From a purely chronobiological point of view associated with the photoreceptive mechanisms, the onset of DST should be delayed until the new condition does not bring significant shares of human activity back to the dark morning. Likewise, the return to the winter setting must be done before the onset times start occurring before sunrise. Figure 1*b* shows the solar altitude at SRW and ${\mathrm{SRW}}^{-}$ for latitude 40°; see also figure S3 in electronic supplementary material. Early April and early October (two weeks after either equinox) seems a fair setting that prevents early risers from experiencing a dark morning around the equinoxes.

The impact of transition dates in the hazards of seasonal DST can be assessed from a natural experiment. Fritz *et al*. [9] analysed the increase of the hazards in US traffic accidents associated with the transition dates from 1996 to 2017. The Energy Policy Act altered transition dates in 2007 enlarging the DST period and narrowing the ST period. Coincidentally, the hazards associated with morning accidents increased by a factor of two [95]. The finding is saying that by setting DST too early, greater shares of human activity are placed in the dark hours of the morning, which leads to a higher number of accidents. A reverse effect was not found in the evening, likely because human activity is not symmetrical. Traffic in morning rush hours could be more intense that in the evening hours, when traffic might be distributed in a larger time interval. Also, it must be taken into account that during the morning rush hours drivers—having just awoken—may not be fully alert, whereas later, for the commute home, they are usually in the middle of their wake.

No study has analysed the impact of the Energy Policy Act on AMI incidence around the spring transition dates, which remains an open question. Janszky & Ljung [10] reported ‘essentially the same results’ when their records of AMI (1987−2006) in Sweden was analysed as a whole, or separately as 1987−1996 and 1997−2006, following the change in the autumn transition date (from late September to late October) that occurred in Continental Europe in 1997.

## 5. Does seasonal daylight saving time misalign human life?

The modern criticism to clock regulations links them to time zones, sees the use of ‘artificial time zones’ or ‘wrong time zones’ as in the following quotes: ‘The issue of DST is an indirect consequence of (...) time zones’ [17]; ‘DST-induce changes are theoretically equivalent to geographical translocations (...) DST translocates the inhabitants of Central Germany to Morocco’ [11]; ‘people in Chicago have to work during the office hours of New York and people in Berlin have the office hours of St Petersburg [12]; and ‘introducing DST is equivalent to making people live according to the local clock time of one time zone further east (i.e. Chicago residents must live on Boston time)’ [96]. By putting forward that the current regulations ‘translocate’ people or people’s time from one place to another, they are presented as inherently artificial and damaging [97]. Indirectly, the sponsors vow for year-round social clocks and year-round social preset schedules, therefore for a year-round phase of human activity, regardless of the seasonal variations.

The modern criticism to DST ignores that clock regulations only ponder *local*, environmental conditions related to the light (summer) and dark (winter) *local* conditions. The practice does not ‘translocate’ residents. It is ‘artificial’ only in the same sense as preset schedules are ‘artificial’: social artefacts that synchronize the phase of human daily rhythms to the sun cycle. As an example: in the case of the 1810 Spanish National Assembly, which of the two preset start times—10.00 in winter, and 09.00 in summer—was ‘artificial’, ‘wrong’ or ‘misaligned with the sun clock’? Certainly, neither of them as they occured in daylight. We note this setting is like 10.00ST in winter and 10.00DST in summer. Inevitably the ‘misalignment’ with the sun clock refers to the ‘misalignment’ with the upper culmination—which is uniform—whereas the timing of the human cycles—specifically the timing of the work cycle—seeks alignment with sunrise times, which do change with latitude and season. Regarding figures 1*a* and 2*a*, and having in mind that the spring–summer morning is alike in New York and in Bogotá, why are New York residents misaligned when, after the spring transition, their start times are like those of Bogotá, while Bogotá residents are not?

A new hazard emerged from this point of view: the long-term or chronic impact of seasonal DST, associated with the long period of time when clocks are set to the ‘wrong time zone’, and people are ‘translocated’ to another place [14,17,97]. Unlike the short-term impact of clock regulations, the long-term impact cannot be assessed from the natural experiment that clock regulations set. On the contrary, currently it is not possible to deconfound the long-term impact from the impact of the shortening of the nighttime (summer) or the shortening of the daylight (winter) [17] or from the impact of modern life.

Johnson & Malow [97, table 1] report ‘chronic DST’ effects that include $20\;\min\;d^{-1}$ ‘sleep loss throughout DST’ from a German-based self-reported study [11]. From the same table another study, Russian based [98], report only $10\;\min\;d^{-1}$ sleep loss with seasonal DST compared with permanent ST, and $4\;\min\;d^{-1}$ sleep loss permanent DST versus permanent ST. Martin-Olalla [52, table S3] reports 20 seasonal differences in sleep times extracted from five time use surveys (in USA, Spain, Italy, France and United Kingdom) and four population groups (employees and non-employees, in weekdays and in weekends). They show very slight differences: average 5min sleep loss in summer, range $15\;\min\;d^{-1}$ sleep loss in summer (French employees in weekends) to $7\;\min\;d^{-1}$ sleep gain in summer (UK non-employees in weekdays); half of the differences were not statistically significant. On the other hand, Monsivais *et al*. [99] conducted a large-scale analysis of mobile phone data in Italy during 2007 and reported that ‘the nocturnal resting period is strongly influenced by the length of the daylight’—therefore by season and latitude. The authors computed the difference between the period of low activity in winter and in summer and reported (figure 3) $25\;\min\;d^{-1}$ loss at 43° latitude and $40\;\min\;d^{-1}$ at 37° latitude.

By contrast, the eighteenth century Swedish case reports approximately $4\;h\;d^{-1}$ less time in bed in summer, due to earlier rise times [49]; and as noted above Yetish *et al*. [37] and de la Iglesia *et al*. [35] reported approximately $1\;h\;d^{-1}$ sleep loss at ϕ=15° and at ϕ=23° in present day, pre-industrial societies. We quote these sleep-loss values as evidence of seasonal variations in the sleep–wake cycle under more naturalistic conditions. A comparison with the modern life reports [11,52,99] is complicated because their smaller sleep loss values can arise from the modern preference for regular schedules and from the modern sleep deprivation, with a marked impact in the winter season: the long winter nights are nowadays less dark than previously. We only note that the chronic sleep loss ‘throughout DST’ (approx. 5% of a daily sleep time at most) seems not to be associated with DST itself.

Another metric that raises concern in this context is the social jet lag (SJL) [100]. SJL reports differences in human sleep timing from work days—entrained to the social life—to work free days—entrained to the circadian clock. Therefore, SJL is associated with modern life and, specifically, with sleep deprivation during weekdays, and has been linked to health issues [101]. It is also strongly dependent on age, with younger population more prone to high SJL [58]. We note that SJL does not aim at the issue of clock regulations—the timing of human activity in workdays—but to weekdays to weekend differences in the timing, which is a derived quantity. Therefore SJL plays a secondary role in assessing the utility of clock regulations. Yet again it is elusive whether potential changes in SJL throughout seasonal DST should be associated with the clock regulations alone, with seasons/latitude or with modern life.

Borisenkov *et al*. [102] conducted a cross-sectional retrospective analysis on continuous data from 2009 to 2016 over children and adolescents (scholars) in Russia (ϕ∼60°), following a natural experiment: Russia went from seasonal DST (2009−2011), to permanent DST (2011−2014), and to permanent ST (since). The authors were able to report sleep timing metrics, including SJL, during the three periods. Borisenkov *et al*. [102 tables 5*a,b* and 6] show worse scores during the permanent DST period (approx. 30min increase in SJL compared with the permanent ST period, as an example), a result which is very much highlighted by the authors and elsewhere [17,18,97]. Interestingly, and much less highlighted, scores for the permanent ST and the seasonal DST showed slight differences (2min⁡ difference in SJL, as an example) suggesting that the impact of seasonal regulations on SJL may be weak. We note that school start times (08.30 local time, reported on [102,table 4] were always before SRW (see electronic supplementary material, figure S4) and that this difference increased during the permanent DST period.

We have not found a study reporting seasonal values of SJL under clock regulations. Roenneberg *et al*. [17] relate SJL to DST as follows: ‘DST increases the discrepancy between the sun clock and the social clock and will therefore also increase the discrepancy between the body clock and the social clock, thereby also increasing SJL’. The association is then based on the rationale presented above: no further study or evidence relating SJL to seasonal DST is provided. The same wording is found in [14] .

The chronic effects of DST are also inferred from studies that report east–west gradients within a time zone, and at time zone boundaries. These are natural experiments in US time zones (cancer prevalence [103,104], societal and health issues [105], accidents [106]), Russian time zones (cancer prevalence [107]), in India (several societal issues [108]), and in China (several societal issues [108–110]).

These research studies are reporting the risks associated with the standardization of time—the use of a common time reference along wide areas, by contrast to the ancient, local mean solar time—because it synchronized human activity along wide areas. With synchronous human activity, longitude or position in a time zone brings late (east) to early (west) human activity relative to the sun clock. Yet, the predictor of these studies is usually the average sunset time [105,108]—which is nothing but a proxy of longitude—highlighting that, unlike wake-up times, bedtimes are not synchronous along a time zone but correlates with the sun clock, which brings late bedtimes in the west side of a time zone. Eventually this results in a sleep loss gradient in the west direction: $19\;\min\;d^{-1}$ discontinuity at the boundary of US time zones (less than 5% of a daily sleep intake), which is then associated with a myriad of societal issues [105]. On the other hand Gentry *et al*. [106], report 20% more vehicle fatality rates in ‘eccentric time localities’, those west of their physical time zone, although Martin-Olalla [111] noted that some confounding factors should have played a significant role in building up this number.

When DST is seen as a change of a time zone towards the east, the above rationale is literally applied, and it is inferred that DST is chronically hazardous [13,14,17,105]. The argument might work if permanent DST and permanent ST are compared—in line with Borisenkov *et al*. [102]. However, the inference is a mistake if the practice of seasonal DST is tested. The issue of seasonal DST is not a comparison of yearly averaged synchronous activity in locations that differ on longitude (the focus of the above studies), but a comparison of non-synchronous activities on the same location and punctuated by seasons: does a convenient start point of activity in winter cease to have application in summer after the sunrise time advanced? [4]; see also the discussion on figure 1.

The hypothesis of a ‘misalignment’ throughout DST is elusive if only because pre-modern human activity should have displayed, under a more naturalistic environment, the seasonal behaviour that characterize many living forms in Extratropical latitudes [39,40]. In §2, we presented a series of studies showing a seasonal change in the timing of human cycles [35,37,48–50,53]. We now note that in these studies the changes are not equally arranged, unlike the changes in the sun cycle: rise times do not come earlier and bedtimes later following early sunrise and late sunset. The above studies often show earlier rise times in summer by 1 h (at 20° latitude), 2 h (43° latitude) or more (60° latitude) with little changes in bedtimes. In addition, we also note that the modern work daily rhythm (see figure 2) is often skewed: a quick morning onset, meaning a narrow distribution of work onset times, by contrast to a long vanishing afternoon offset, signalling a wider distribution of work offset times. Clock regulations went in line with these observations and focused on accommodating earlier onset times during the DST period.

Should the phase of the general population be ‘misaligned’ during the summer season under seasonal DST, then the regulations would have not sustained in time or people would have acted by delaying their preset time schedules in summer to offset the ‘misalignment’. However, after 100 years of clock regulations, this behaviour is not observed when winter daily rhythms and summer daily rhythms extracted from time use surveys are contrasted [52, fig. 2]. To be more specific, throughout DST people do not delay their wake-up hour of the day relative to the wake-up hour of the day throughout ST [52,112]. Kantermann *et al*. [11, fig. 1], Zerbini *et al*. [113, table 1], a Dutch-based study and Sani *et al*. [47, fig. 4], an actigraphy study in Chicago, also show this lack of response. These studies are key evidence that clock regulations are operating fine, aside from the issues associated with transition dates [2, p. 16].

Adaptive responses to clock regulations are indeed known in history. When Spain (mainland) remained in the Central European Time (UTC+1) after World War II, 1 h ahead of the sun clock, social preset schedules continued to entrain to the sun clock, meaning that they apparently ‘delayed’ by clock time. This response is still surrounded by confusion to many [17,114]. When permanent DST was tested in United Kingdom and in Ireland (1970), in United States (1973), in Portugal (1967), in Russia (2011) or in Chile (2015) the setting did not sustain due to the discomfort brought by start times before SRW. When Portugal (1992) advanced its clocks by 1 h year-round to align them with Central European Time; the setting survived only 3 years and, seemingly, caused a political crisis.

No such adaptive responses are known regarding the application of summertime during the spring, summer seasons in Europe and America. On the contrary, the inception of clock regulations and their long sustainability can be understood as a responsive action against the discomfort that start times aligned to SRW brought in the spring, summer seasons.

Finally, there is one chronic side effect of the clock regulations that must be considered for a proper balance of the pros and cons of the practice. By promoting early activity only in summer, and by delaying activity in winter, clock regulations have played, and play, against the incorporation of human activity into the dark hours of the winter morning, now possible with the use of artificial light [115]. Start times in United Kingdom, where clock regulations have applied since 1916, are better aligned with SRW than counterparts in Germany, where clock regulations were not used from 1947 to 1980. This phase delay can be a positive outcome following the east–west gradient studies [18,105].

## 6. Why did seasonal daylight saving time succeed?

More than a century ago informed people tried to educate others about the wonders of the seasonal changing of the clocks [4,116].

The sponsors of seasonal DST promoted a bimodal human activity in Extratropical societies synchronized by preset schedules that mimicked pre-modern, seasonal human activity, albeit imperfectly. Hudson [3] addressed the role of latitude limiting the practice to countries beyond 30° latitude, and to seasons. Hudson [4] set the rationale of the practice: ‘In order to more fully utilise the long days of summer’ so that ‘a long period of daylight leisure would be made available in the evening for (...) any (...) outdoor pursuit desired’. The rationale follows the rule of thumb ‘duty first before pleasure’, which does not conciliate with the symmetry of solar activity: the increase in daylight is driven by even shifts in sunrise and sunset times.

Some medical associations also supported the practice. The first mention to seasonal DST in the *Journal of the American Medical Association* reads [117]: ‘Urging the need of more light, air and sunshine, the Dutch society for the progress of medicine has petitioned (...) the adoption of daylight-saving time. The point of issue is an amelioration of living conditions rather than a question of economy.’ Likewise, DST started in Italy (1964) accompanied of remarks on the psychological improvements [118].

While the impact of the changing of the clocks in health issues is slight, limited to specific issues, and confounded among many other external factors (see §§3 and 5), the impact on the original goals is noticeable as morning activities align closer to the sunrise. All else equal, the range of the time distance from preset start times to sunrise times is shortened from 3h to 2h—a 30% reduction—when clock regulations apply at 40° latitude. Sunrise ambient light then keeps as a cue for onset times, from wake-up to the start of work. figure 2 (left bottom) visualizes the case in Italy.

On the other part of the day, individuals quickly perceived the improvement in leisure hours: early work onset leads to early work offset and longer daylight leisure evenings during the spring and summer. If the day is evenly broken in sleep, work, and leisure, 1 h makes 12.5% of the available leisure time. At this point, we should also recall the dramatic increase in leisure time that the twentieth century brought [119]. Furthermore, with work onset aligned to SRW in winter and ${\mathrm{SRW}}^{-}$ in summer bedtimes do not easily come before SSS in summer, see again figure 3 (left bottom).

## 7. Why has permanent standard time proven hard to implement?

Most of the current surveys show larger shares of population choosing permanent DST over permanent ST [20]. While Roenneberg *et al*. [17, Myth-understandings and confusions surrounding DST] associate this preference with the bias ‘summer’ versus ‘winter’, we note that this choice is showing that newer generations still accommodate with the current summer setting, and their abhorrence for a delay that permanent ST would bring. It is further evidence that the setting designed by Hudson [4] does not play against human preferences [26].

Compared with early twentieth century, informed people, chiefly sleep associations, are nowadays trying to educate others of the merits of locking the clock, and, specifically, the wonders of returning to a solution (permanent ST) that was abandoned decades ago. They are promoting a unimodal, non-seasonal human activity that poorly resembles the seasonal cycles of behaviour [39,40] that should have been present in pre-modern, Extratropical human activity. If energy saving prompted governments to act during the twentieth century, now it is the fear that the hazards might be worse than expected that is prompting action. Fear has always been a compelling argument.

While locking the clocks may look fine to anyone, the problems arise soon afterwards because the circadian clock, the mechanical clock, and the preset schedules—the three of them prone to a fixed 24h period—cannot conciliate well with the timing of light and dark at midlatitudes, which does not observe this period. Then, which clock should survive? Or, in other words, which hour of the day is best to start the day? That hour people are used to in summer, or that hour people are used to in winter? An intermediate hour of the day? Should people delay the activity in summer, when the sunrise advances most, or should people advance the activity in winter, when the sunrise delays most? The answer to these questions plays against human physiology, like deciding whether to wear sandals in winter, or boots in summer [120].

The sponsors of permanent ST know beforehand of these issues. In this line, we note two points. First, Roenneberg *et al*. [17, Potential solutions] suggest: ‘anyone who wants to spend more time at home in daylight after work should convince his/her company and co-workers to advance their time during certain months’. In other words, governments should lock the clocks, and, in return, allow the seasonal changing of preset schedules, which is nothing but the same. Eventually, the issues at stake are now a matter of preferences, and not a serious health or societal issue. The irony here is that, currently, individuals can also convince their co-workers to offset the spring clock change with a delay of their schedules, should they want to spend less time at home in daylight after work. This demand is virtually unknown, as per data extracted from time use surveys [52]; however, the call for permanent ST is pushing in this direction.

Second, Malow [13, Splitting the difference?] describes another choice for USA: a permanent clock set at 30min ahead of ST and at 30min behind DST. Understandably Malow finds it inadequate from a logistical perspective due to off synchronization with global partners, and from the usual circadian arguments. Nonetheless, this option is worthy to note because, on the one hand, it highlights the need of a practical compromise between those who prefer ST and those who prefer DST.

On the other hand, this specific compromise fits the current yearly average time zone, which is, roughly, ST+30min. However, it loses synchronization with seasons. By getting rid of the annoying changing of the clocks, it finds a year-round annoying starting point, with darker winter morning hours (albeit shorter than permanent DST), and brighter summer morning hours (albeit less bright than permanent ST). From an historical point of view, this compromise is also an interesting try since both permanent ST and permanent DST failed to sustain in USA.

The push for permanent DST—and for permanent ‘half-DST’—finds a narrative when comparing the early twentieth century with the early twenty-first century. During the twentieth century, artificial light has prompted many people to advance the start of the day shifting their start times from SRW to earlier hours, thus harvesting dark hours of the winter dawn, which plays against human physiology. In the context of clock regulations, the harvesting of these hours is the most distinctive difference when the scenario at the beginning of the twentieth century is compared with the scenario at the beginning of the twenty-first century.

The harvesting of the dark hours of the winter dawn is also promoted by latitude because above approximately $50^{\circ }$ latitude daytime is shorter than the usual work hours (see figure 2*b*), and people find new benefits with this choice: leaving work before the sun sets in winter [43]. Figure 3 (top right) visualizes the extreme scenario in Finland, where daytime lasts some 5h. Human activity in winter resumes before sunrise during a long twilight period, and starts fading away just at winter dusk. With work onset occurring at ${\mathrm{SRW}}^{=}$ bedtimes come before sunset in summer. Eventually, above some circle of latitude sunrise stops cueing efficiently human activity. We note that sunrise at 61° latitude spans 5h from winter to summer: the 1-h shift that clock regulations bring has a lesser impact than at 40° latitude. This would explain why high latitude countries and regions are opposing seasonal DST and some of them—Iceland (1969), Russia (2011), Saskatchewan (Canada, 1961) and Región de Magallanes (Chile, 2017)—stopped practicing it. We note that they prefer early winter activity instead.

## 8. Concluding remarks

We have revisited the link between seasonal clock regulations and human physiology (photoreceptive mechanisms), and put a context to the modern criticism against the regulations. Unlike many other scientists in this field, we do not have a bold concluding statement other than acknowledging that clock regulations played, and will play a useful role in regulating the timing of human activity at Extratropical societies synchronized by clocks.

We do not support clock regulations to continue globally. We do not support that clock regulations *must* come to an end globally either. Instead, we provide a rationale under which clock regulations have succeeded and stress that many confounding factors, including cultural and environmental, impact whether a given society would love or would abhor seasonal clock regulations. In this line, we understand the recent calls to end regulations in Mexico, Brazil, Russia and Región de Magallanes (Chile), all of them related to the impact latitude (low or high) in human daily life. On the contrary, we put forward that the hard times that American and European decision-makers have found when addressing the cancelling of the clock regulations are evidence of their utility and of the benefits that many get from them, usually inadvertently. Regarding the European Union, and taking into consideration its wide range of latitude (35° to 60°), we are sceptical that a uniform policy on summertime arrangements be sound.

We maintain that with increasing latitude there is a pressure to delay (the phase of) human activity in winter, associated with the delay of sunrise times and to our photoreceptive mechanisms that set alertness. We sustain that this stressor ceases in spring–summer; therefore, a pressure to restore an earlier (phase of) human activity in summer emerges associated with the advance of the sunrise times (hence, still dominated by our photoreceptive mechanisms), with the high insolation at noon, and with a social rule of thumb—‘duty first, before pleasure’. We do acknowledge that there is also a pressure to sustain regular preset schedules, associated with our circadian 24h system that loves a regular, uniform daily rhythm.

Seasonal clock regulations are only one synchronized way of solving these conflicting processes. Their main drawback continues to be the issues associated with transition dates, which disrupt the regular social daily rhythm. However, we do not see any relevant, specific hazard associated with transition dates. The short-term increase of risks in accidents or in myocardial infarction is slight (*z*-score equal to 1/3) compared with the myriads of confounding factors. If clock regulations were associated only with energy saving, then any increase in the risks would be unacceptable. However, since clock regulations are associated with human physiology, then we must admit that they are just one of the many risks that face modern, complex, synchronized societies like the use of uniform time in wide areas and the use of uniform preset schedules. The key question to address is whether one specific hour of the day can sustain as a year-round starting time in view of the changes in sunrise times that latitude brings.

We maintain that much of the current discussion is focused on the phase (timing) of human activity in summer because its extended daytime allows different layouts for human activity without challenging key aspects of human physiology. While sleep associations would love delayed times in summer, many still love the current setting and enjoy their longer leisure time in daylight. We do not exclude that a delay of the phase in summer might yield benefits in some key health issues, yet the lack of adaptative responses and the fact that morning, pre-modern, ancient activity was probably like the current setting, suggest that these benefits might be marginal. Seasonal clock regulations by themselves do not play against human physiology.

The phase (timing) of human activity in winter is less prone to change because the short daytime makes it difficult. Nonetheless during the twentieth century and beyond, with the advent of cheap and efficient artificial light, many Extratropical societies have harvested the dark hours of the winter dawn reducing the phase delay that latitude brings. This decreases the necessity of a seasonal change in spring. We note that clock regulations come to an end when the winter phase delay of human activity is reduced.

Based on the ambient light conditions at the start of the day, we suggest early April and early October as the appropriate transition dates, matching with current dates in Australia and New Zealand. The extension of DST to October in Europe and in America might bring benefits to sectors linked to outdoor activities, but challenges for no good reason those with start times at dawn—the early risers, including scholars: daytime is long enough in October to accommodate a work onset in daylight.

We maintain that the best indicator to assess the utility of seasonal clock regulations is the relation between the starting point of human activity and the winter sunrise time. Daily rhythms extracted from time use surveys provide enough evidence to assess sleep offset and work onset on a given society. Then these time marks must be compared with SRW. No hard rules exist on this topic but societies whose start times are in line with SRW would love seasonal clock regulations. Those societies with start times before SRW would be less keen of seasonal clock regulations. Further studies are needed to confirm this hypothesis. The results from 2018 European Commission public consultation on summertime arrangements might provide a first evidence [54].

## Data Availability

This article has no additional data. Supplementary material is available online [121].

## Footnotes

^1^
The rate of events following the transition over the rate of events prior to the transition.
